# Reductive carboxylation mediated oxidative stress defense supports anchorage independent cell growth

**DOI:** 10.1186/2049-3002-2-S1-P30

**Published:** 2014-05-28

**Authors:** Lei Jiang, Matthew Mitsche, Ralph Deberardinis, Karine Smans

**Affiliations:** 1 Children’s Medical Center Research Institute, UT Southwestern Medical Center, Dallas, TX, USA; 2 Eugene McDermott Center for Human Growth and Development, UT Southwestern Medical Center, Dallas, TX, USA; 3 Oncology, Janssen Research & Development, Beerse, Belgium

## Background

Cancer cells consume large amount of glutamine for growth and proliferation. In normal mitochondria, glutamine is converted to α-ketoglutarate, which is further oxidized through the TCA cycle [1]. Recent studies find that under certain extreme conditions, such as mitochondria dysfunction and hypoxia, cells use glutamine through reductive carboxylation (RC) to generate acetyl-CoA for lipogenesis. During tumor development, the acquisition of anchorage independence enables cancer cells to survive without their natural extracellular matrix, but this comes at the price of increased oxidative stress [2]. Here we examined reprogramming of glutamine metabolism during adaptation to growth of anchorage independent tumor spheroids, including reprogramming of pathways that counteract production of reactive oxygen species (ROS).

## Materials and methods

Metabolic differences were compared in cancer cells cultured under monolayer adhesion and suspension spheroid condition. ^13^C labeled glucose or glutamine cultured cells were extracted for metabolites, followed by gas chromatography mass spectrometry analysis.

## Results

Here we show that spheroid culture is associated with an induction in mitochondrial ROS. Under these conditions, cells prefer to use cytosolic RC rather than the intra-mitochondria oxidative pathway to initiate the glutamine metabolism. Cytosolic isocitrate dehydrogenase 1 (IDH1) is the only isoform of IDHs catalyzing RC in spheroid culture. Blocking the cytosolic RC with an IDH1 specific inhibitor induces mitochondrial ROS, and impairs spheroid growth, which could be rescued by antioxidant N-Acetyl Cysteine (NAC) co-treatment. ^13^C labeling in isolated mitochondria reviewed the import and oxidation of citrate, using a pathway that may result in the transfer of NADPH from cytosol to mitochondria.

## Conclusions

Our finding indicates that IDH1 dependent reductive carboxylation incorporates cytosolic reducing equivalent into citrate, which gets into the mitochondria and releases the reductive equivalent as NADPH through the oxidative TCA cycle. These results reveal a novel reductive carboxylation mediated NADPH transportation pathway, to protect cells from detachment induced mitochondria oxidative stress.
